# Morphological and Functional Evaluation of Oral Citicoline Therapy in Chronic Open-Angle Glaucoma Patients: A Pilot Study With a 2-Year Follow-Up

**DOI:** 10.3389/fphar.2019.01117

**Published:** 2019-09-26

**Authors:** Michele Lanza, Ugo Antonello Gironi Carnevale, Luigi Mele, Mario Bifani Sconocchia, Silvia Bartollino, Ciro Costagliola

**Affiliations:** ^1^Multidisciplinary Department of Medical, Surgical and Dental Specialities, Università della Campania “Luigi Vanvitelli”, Napoli, Italy; ^2^Department of Medicine and Health Sciences “V. Tiberio”, University of Molise, Campobasso, Italy

**Keywords:** glaucoma, citicoline, neuro-enhancement, neuro-protection, OCT, visual field

## Abstract

**Aims:** To study the neuroprotective effect of oral citicoline (CT) therapy in primary open-angle glaucoma (POAG).

**Methods:** This study included one eye each of 60 POAG patients. Patients were randomly divided into two groups (A and B) of 30 participants each. Only patients of group A were administered with CT therapy. Age, sex, and disease duration were matched between groups. Despite a stable intraocular pressure (IOP), a slow disease progression—assessed by standard automated white-on-white perimetry (SAP) in the previous 3 years—occurred in all patients. All patients underwent a complete eye examination, including IOP measurement, SAP, retinal nerve fiber layer (RNFL) thickness, and ganglion cell complex (GCC) thickness measurements with optical coherence tomography (OCT), before starting CT treatment and at 6, 12, 18, and 24 months’ follow-up. Parameter differences between groups were evaluated at each eye check.

**Results:** After 18 months, mean values of SAP mean deviation (MD) of group A were significantly (*p* = 0.039) higher (−7.25 db) than those of group B (−8.64 db). Moreover, they appeared stable in the following visits, whereas in group B, mean MD values continued to significantly (*p* < 0.001) decrease (−9.28 db) over time. Mean RNFL and GCC thickness in group A were significantly (*p* < 0.01) higher (70.39 and 71.19 μm, respectively) than in group B (64.91 and 65.60 μm, respectively) after 12 months of CT therapy. Furthermore, they appeared to be stable over the later visits, whereas they thinned significantly (*p* < 0.001) over time in group B.

**Conclusion:** These findings suggest that CT therapy seems to be effective in slowing POAG progression. Further studies on a larger population and with a longer follow-up are needed to confirm this pilot investigation.

## Introduction

Glaucoma is an optic neuropathy characterized by the thinning of the retinal nerve fiber layer (RNFL) and an increase of optic disc cupping (Quigley et al., 1989; Weinreb and Khaw, 2004). It is one of the leading causes of irreversible blindness, if not properly detected and managed (Kingman, 2004). The therapeutic strategy used by ophthalmologists for primary open-angle glaucoma (POAG) is to lower intraocular pressure (IOP) by using drugs, laser, or surgical therapy (AGIS, 2000; Heijl et al., 2002; Maier et al., 2005; Foganolo and Rossetti, 2011). Despite a significant reduction of IOP, many cases defined as “well controlled” show a disease progression (Sommer, 1989). Moreover, it has been demonstrated that glaucoma not only affects the optic nerve but also involves all the visual pathways, causing, in the later stages, changes in geniculate lateral nucleus (GLN) and visual cortex (Gupta et al., 2006). These considerations have led or lead to new definitions of glaucoma and to new perspectives in POAG therapy (Gupta and Yucel, 2007). Neuroprotection represents a new chapter in the disease treatment, intended to preserve structures and functions of the visual system (Rejdak et al., 2003; Tian et al., 2015). Several molecules have neuroprotective properties, and, among them, citicoline (CT) appears to be very interesting and has been tested in different studies (Oshitari et al., 2002; Yücel et al., 2003; Saver, 2008; Silveri et al., 2008; Tian et al., 2015).

CT is a nucleotide formed by ribose, cytosine, pyrophosphate, and choline, which play a crucial role in the synthesis of phospholipids, particularly glycerophospholipid phosphatidylcholine (Secades and Lorenzo, 2006; Silveri et al., 2008). Phospholipids are the main components of the cell membrane, as they guarantee the structural integrity of cells and are the fundamental compound for several enzymes (D’Orlando and Sandage, 1995). After oral administration, CT is hydrolyzed, in the intestinal wall, to choline and cytidine, and then the latter is converted to uridine (D’Orlando and Sandage, 1995; Saver, 2008; Wignall and Brown, 2014). After absorption, these compounds reach the central nervous system (CNS) where CT can be restored by the CTP-phosphocholine cytidylyltransferase enzyme (D’Orlando and Sandage, 1995; Grieb and Rejdak, 2002; Saver, 2008; Wignall and Brown, 2014).

Glaucomatous patients present neural transmission delay in the GLN (Oshitari et al., 2002; Yücel et al., 2003). The afferent axon of retinal ganglion cell releases neurotransmitters in the inter-synaptic space, activating the GNL’s cells, which in turn develop the ganglion cell’s axon, relaxing neurotrophic factors (Weinreb et al., 2014). A trans-synaptic dysfunction at this level is a consequence of retinal ganglion cell loss in glaucoma (Gupta and Yucel, 2007). CT acts on this dysfunction through its neuroprotective and neuro-enhancement properties, thanks to both its neuro-modulatory action on the dopaminergic system and the synthesis of phospholipids (Secades and Lorenzo, 2006; Saver, 2008). Three previously published papers have shown that in glaucoma patients, CT treatment improves the retinal bioelectrical responses and the activity of the visual cortex, slowing down the glaucomatous rates of progression (Parisi et al., 2008; Ottobelli et al., 2013; Parisi et al., 2015).

Herein, we evaluate both functional [standard automated white-on-white perimetry (SAP)] and morphological [optical coherence tomography (OCT)] parameters in glaucomatous patients treated with orally administered CT.

## Methods

This prospective study included 84 glaucoma patients randomly divided into two groups (groups A and B), with similar age, sex, disease duration, and disease stage, who had been referred to the Glaucoma Unit of University of Molise, Campobasso (Italy), on starting neuroprotective co-adjuvant treatment. The study was performed in accordance with the ethical standards stated in the 1964 Declaration of Helsinki and approved by the local clinical research ethics committee. Informed consent was obtained from all subjects after being given a detailed description of the objectives of the study and the procedure to be used. Investigations were conducted in accordance with Good Clinical Practice (GCP). POAG diagnosis and classification were based on the Glaucoma Staging System (GSS) (Brusini and Filacorda, 2006). Patients selected for this study had shown a SAP mean deviation (MD) reduction ranging between 1 and 1.5 db/year during the previous 2 years, although IOP had apparently stablilized with therapy [≤18 mmHg measured with Goldmann applanation tonometry (GAT)]. All patients underwent a complete eye examination, including IOP measurement with GAT, gonioscopy, ophthalmoscopy, central corneal thickness (CCT) measurement with a Scheimpflug camera-based device (Oculus Pentacam, Wetzlar, Germany), SAP, and RNFL and ganglion cell complex (GCC) evaluation with OCT (RTVue, Optovue, Freemont, CA, USA). GAT was always performed in the afternoon, between 2 p.m. and 4 p.m.; SAP was performed with a Humphrey Field Analyzer (HFAII, Carl Zeiss Meditec, Dublin, CA, USA), with a size III stimulus, Swedish interactive threshold algorithm (SITA) standard, and 30-2 pattern. Each glaucoma patient referring to both units underwent SAP every 6 months, so they were used to the procedure. Moreover, only exams with good reliability indices (with less than 33% fixation losses or false-negative errors, or less than 15% false-positive errors) were included in the study for statistical evaluation. To reduce operator-related bias, physicians were not aware of any subsequent CT treatment. Lowering topical therapy included 0.5 mg of timolol (11, 37%), travoprost 0.004% (10, 33%), and fixed combination of 0.3 mg of bimatoprost and 0.5 mg of timolol (9, 30%). During the follow-up period, no IOP spikes occurred, with a mean IOP increase of no more than 3 mmHg at every check (**Table 1**).

**Table 1:** Means and standard deviation (SD) of intraocular pressure values in groups A and B during the evaluated follow-up with statistical evaluation of differences.

	Baseline	6 months’ follow-up	*p* value	12 months’ follow-up	*p* value	18 months’ follow-up	*p* value	24 months’ follow-up	*p* value
Group A									
Mean (mmHg)	13.83	14.1	0.34	13.6	0.19	14.33	0.11	14.07	0.43
SD	1.34	1.09		1.57		1.37		1.2	
Group B									
Mean (mmHg)	14.3	14.0333	0.37	14.08	0.23	14.2	0.64	14.3	0.41
SD	1.15	1.25		1.315		1.3		2.04	
*p* value	0.15	0.82		0.21		0.63		0.59	

**Figure 1:**
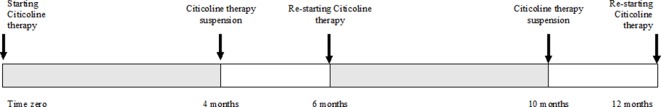
Citicoline therapy cycles for 1-year treatment.

Included patients were randomly divided into two groups: Group A received CT oral solution (Neukron Ofta; Omicron, Rome, Italy), whereas group B did not receive any oral treatment. CT posology was one vial (500 mg of CT in 10 mL of oral solution) per day for 4 months, followed by a 2-month interruption, after which the therapy cycle was repeated again for another 6 months, as suggested by the manufacturer; this cycle of administration is displayed in **Figure 1**. Exclusion criteria were as follows: inability to perform SAP, best corrected visual acuity (BCVA) worse than 20/40, significant ocular media opacities, a history of previous glaucoma surgery, cataract or retinal surgery, and concomitance of ocular diseases that might bias SAP performance or results. Concomitance with systemic diseases that might lead to visual acuity damage or affect SAP execution, low quality (<35) in OCT scans, contraindications, and/or intolerance to CT were also considered as exclusion criteria. Lastly, patients with pseudoexfoliative glaucoma and/or pigmentary glaucoma were also excluded.

Every 6 months, both groups underwent a complete eye visit, SAP, and OCT scans. In addition, in group A, CT therapy adherence and any side effects were evaluated at each check. At the beginning of the study, each group consisted of 42 patients. During the follow-up, some patients had been excluded because of interruptions of the neuroprotective treatment, changes in IOP-lowering therapy, or the need for ocular surgery or they were lost to follow-up. Only the data of patients of both groups who completed the 2-year evaluations without meeting any exclusion criteria were compared and statistically analyzed at 6, 12, 18, and 24 months’ follow-up.

Demographic characteristics and values obtained by SAP and OCT in both groups of participants at baseline are summarized in **Table 2**. GSS 2 classification of eyes included in the study is summarized in **Table 3**.

**Table 2:** Group parameters at baseline.

Parameter	Group A		Group B		P
	Mean	SD	Mean	SD	
Age (years)	64.1	5.8	62.9	7.2	0.465
Sex ratio (M/F)	16/14		16/14		
Disease duration (months)	38.73	1.53	35.53	1.65	0.929
MD (dB)	−6.51	2.65	−6.39	2.03	0.850
RNFL (µm)	72.9	7.3	73.3	4.9	0.785
RNFL Sup (µm)	86.2	7.3	87.4	5.9	0.515
RNFL Inf (µm)	94.6	7.5	95.4	6.9	0.673
RNFL Nas (µm)	70.8	8.8	68.7	6.5	0.305
RNFL Temp (µm)	68.1	8.9	66.3	6.5	0.373
GCC (µm)	73.8	7.5	74.6	5.2	0.626
GCC Sup (µm)	75.6	7.7	75.9	5.4	0.839
GCC Inf (µm)	71.9	8.0	73.2	5.3	0.457

Statistical comparison of demographical data and optic nerve parameters in group A (patients assuming citicoline) and group B (controls), before starting therapy with citicoline. MD, mean deviation measured with standard automatic perimetry (SAP); RNFL, overall retinal nerve fiber layer thickness; RNFL Sup, superior retinal nerve fiber layer thickness; RNFL Inf, inferior retinal nerve fiber layer thickness; RNFL Nas: nasal retinal nerve fiber layer thickness; RNFL Temp, temporal retinal nerve fiber layer thickness; GCC, overall ganglion cell complex thickness; GCC Sup, superior ganglion cell complex thickness; GCC Inf, inferior ganglion cell complex thickness measured with ocular coherence tomography (OCT); p: Student t-test significance.

**Table 3:** Staging of the eyes included in the study.

Glaucoma staging system 2	Group A		Group B	
	Eyes	%	Eyes	%
Generalized defect stage 1	3	10	7	23
Generalized defect stage 2	6	20	6	20
Generalized defect stage 3	1	3	1	3
Mixed defect stage 1	4	13	2	7
Mixed defect stage 2	9	30	7	23
Mixed defect stage 3	6	20	7	23
Localized defect stage 1	1	3	0	0

Classification of eyes included in the study, in groups A and B, according to Glaucoma Staging System 2.

Parameters evaluated were as follows: MD and standard deviation (SD) measured with SAP; RNFL total thickness (RNFL); superior (RNFL Sup), inferior (RNFL Inf), temporal (RNFL Temp), and nasal (RNFL Nas) quadrant thickness; and GCC overall thickness (GCC), superior (GCC Sup), and inferior (GCC Inf) hemifield thickness measured with OCT. Even if every patient had bilateral POAG, only one eye per patient was randomly included in the statistical analysis, in order to reduce intra-subjective bias.

## Statistical Evaluation

The fulfillment of the data requirements for parametric analysis (normality and homogeneity of variance) was assessed by specific tests (Kolmogorov–Smirnov and Levene). For all parameters, a preliminary separate two-way repeated-measures analysis of variance (ANOVA) was carried out, with treatment as between-group factor and time as repeated-measures factor. Based on significant results for time effect (*p* < 0.001) and time × treatment interaction effect (*p* < 0.001) of previous analysis, groups A and B were compared with one-way factorial ANOVA for each parameter. In addition, intra-group time variation of parameters was evaluated with Student’s *t*-test for repeated measures. Finally, the correlations between baseline measures and their variations after 24 months were evaluated using the parametric (Pearson) test. For all tests, the level of significance was set at *p* < 0.05. All analyses were performed using SPSS software (IBM Corp. Armonk, New York) version 18.0.

## Results

At the beginning of the study, the IOP values of group A, measured with GAT, always between 2 p.m. and 4 p.m., ranged between 12 and 16 mmHg (mean: 13.83 ± 1.34 mmHg), and the IOP values of group B ranged between 12 and 16 mmHg IOP (mean: 14.3 ± 1.15 mmHg). After 2 years, the complete data record referred to 30 patients for each group; nine patients were excluded because of interruption of CT treatment and eight because of changes in IOP-lowering treatment, and seven were lost to follow-up.

**Figure 2:**
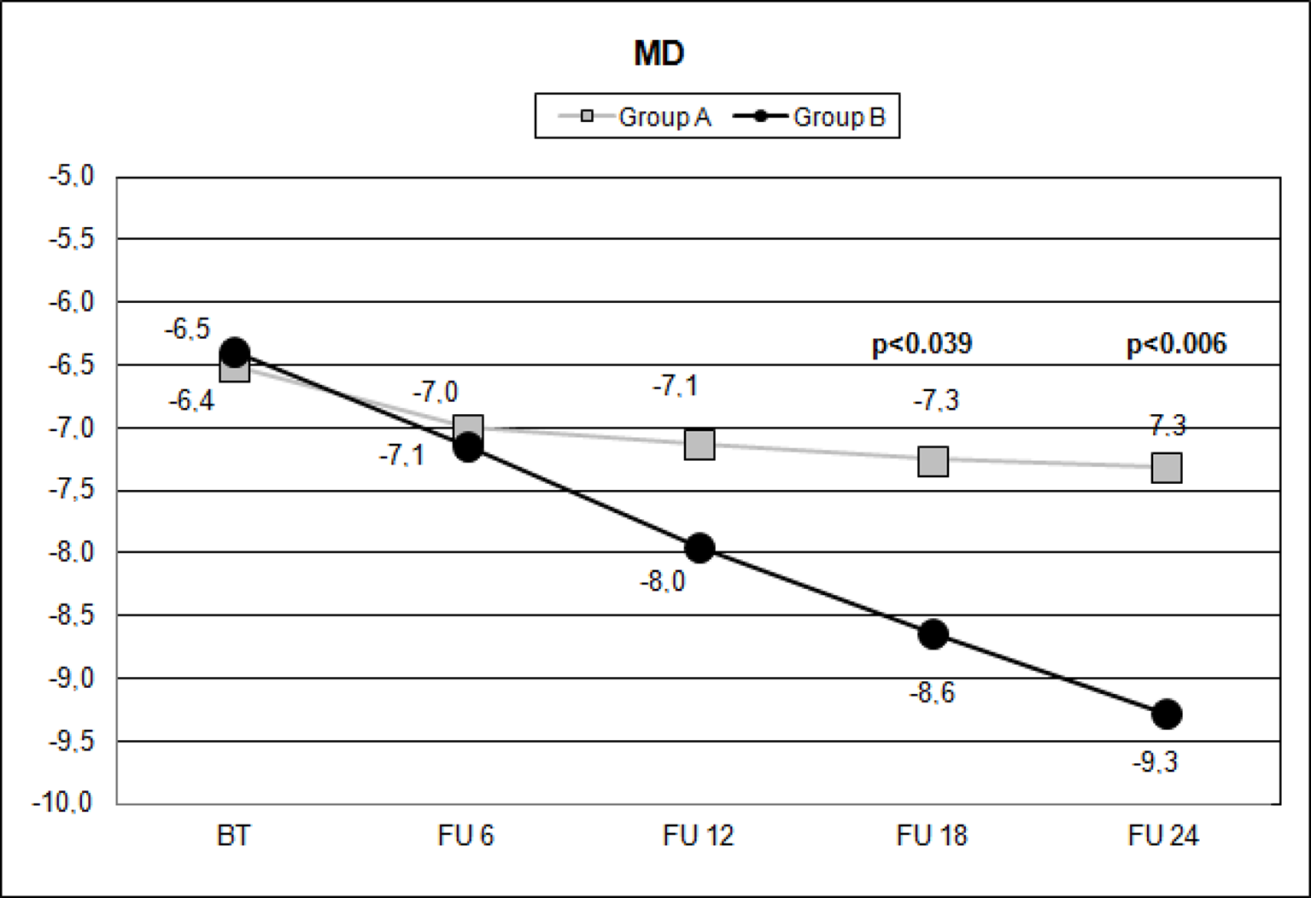
Scatterplot showing mean deviation (MD) values measured (decibel) with standard automated white-on-white perimetry (SAP), on the vertical axis, in patients assuming citicoline (gray boxes) and in patients not undergoing therapy with citicoline (black circle), before starting therapy (BT), at 6 months’ follow-up (FU 6), at 12 months’ follow-up (FU 12), at 18 months’ follow-up (FU 18), and at 24 months’ follow-up (FU 24). *p* values mean significant differences (*t*-test).

**Figure 3:**
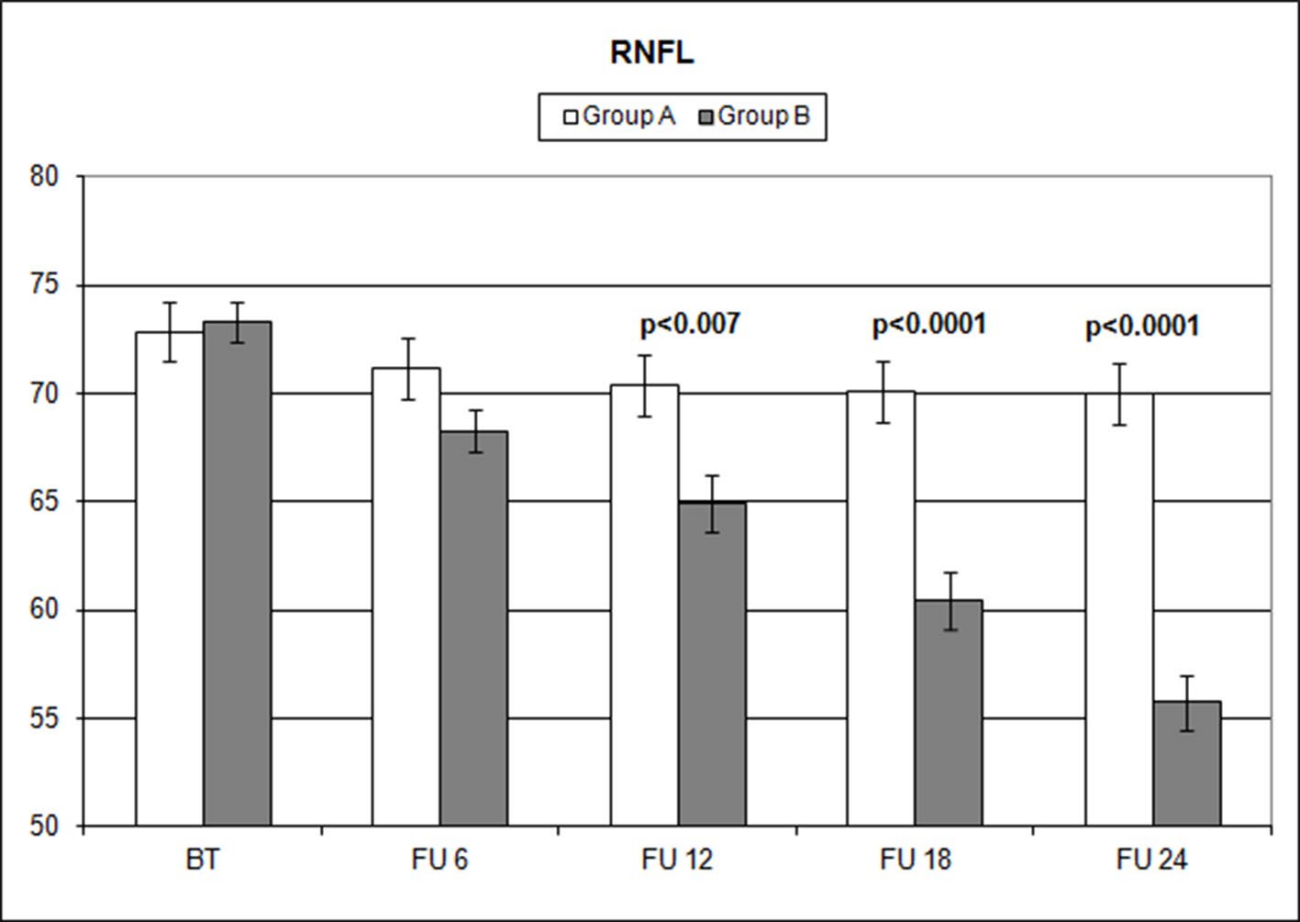
Comparison of overall retinal nerve fiber layer (RNFL) thickness values measured (microns) with ocular coherence tomography (OCT), on the vertical axis, in patients assuming citicoline (white column) and in patients not undergoing therapy with citicoline (grey column), before starting therapy (BT), at 6 months’ follow-up (FU 6), at 12 months’ follow-up (FU 12), at 18 months’ follow-up (FU 18), and at 24 months’ follow-up (FU 24). *p* values mean significant differences (*t*-test).

**Figure 4:**
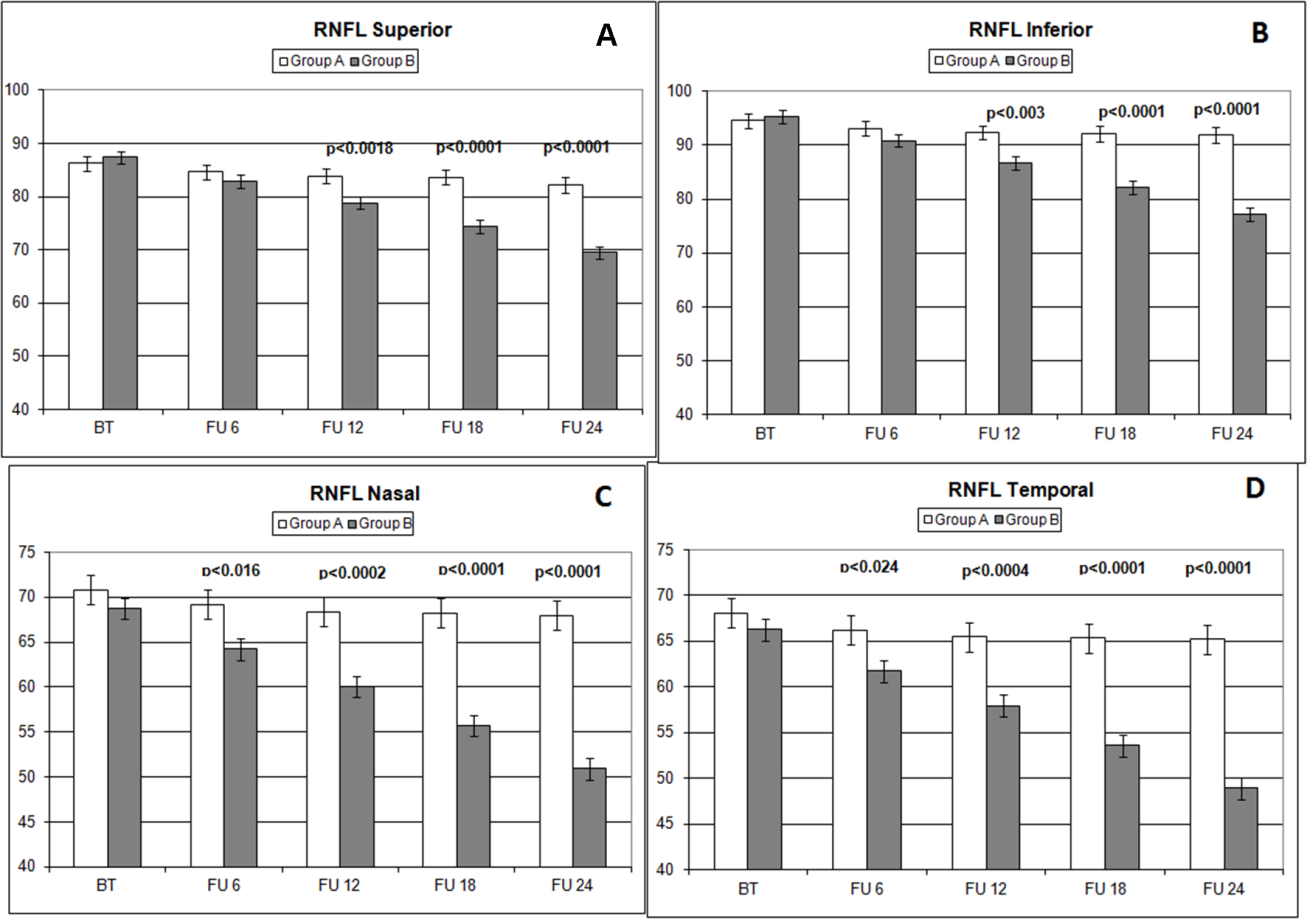
Comparison of RNFL thickness values measured (microns) in different sectors (**A**: superior; **B**: inferior; **C**: nasal; **D**: temporal) with ocular coherence tomography (OCT), on the vertical axis, in patients assuming citicoline (white column) and in patients not undergoing therapy with citicoline (grey column), before starting therapy (BT), at 6 months’ follow-up (FU 6), at 12 months’ follow-up (FU 12), at 18 months’ follow-up (FU 18), and at 24 months’ follow-up (FU 24). *p* values mean significant differences (*t*-test).

Over the follow-up period, patients in group A showed significantly higher values of MD measured with SAP than did those recorded in patients of group B (at 18 months, *p* < 0.039; and at 24 months, *p* < 0.006) (**Figure 2**). Overall RNFL thickness values measured in group A were statistically higher than those in group B at 12 (*p* < 0.007), 18 (*p* < 0.0001), and 24 months (*p* < 0.0001) of follow-ups (**Figure 3**). By analyzing RNFL thickness variations in more detail, it is possible to observe that superior and inferior zones showed statistically higher values in group A eyes compared with group B after only 12 months’ follow-up. In contrast, in nasal and temporal sectors, the differences between RNFL thickness measured in both groups showed statistically higher values in group A eyes starting at 6 months’ follow-up (**Figure 4**). In addition for GCC, it was possible to observe that, overall, both superior and inferior thickness showed significantly higher values at 12 (*p* < 0.003), 18 (*p* < 0.0001), and 24 months (*p* < 0.0001) of follow-ups (**Figure 4**). In group A, fewer variations of all parameters at each follow-up were recorded, and these variations were statistically less significant than those observed in group B (**Table 4**). In group A, only a slight correlation between MD and RNFL values before starting the study and their variations observed during the following 24 months was observed. In group B, each evaluated parameter—except for GCC Inf—showed a statistically significant correlation between values measured at baseline and the decrease observed during 2 years’ follow-up (**Table 5**). Altogether, these data suggest that in group A, the CT treatment effect did not depend on the stage of the disease, whereas in group B, the eyes showed that the occurrence or not of improvement was related to the stage of the disease. No side effect of CT treatment was reported during the whole follow-up period. No statistical difference (*p* = 0.49) in CCT, measured at the start of the study, was observed between group A eyes (mean: 523.73 ± 8.4 µm) and group B eyes (mean: 525.07 ± 6.14 µm).

**Table 4:** Parameter analysis of both groups over the follow-ups.

Paired Student’s *t*-test comparison							
Parameter	Follow-up comparison	Group A			Group B		
		Variation	*T*	*p* <	Variation	*t*	*p* <
MD	0 vs. 6 months	−0.496	12.690	**0.001**	−0.751	12.028	**0.001**
	6 vs. 12 months	−0.131	1.620	0.116	−0.816	12.827	**0.001**
	12 vs. 18 months	−0.119	2.200	**0.036**	−0.682	10.784	**0.001**
	18 vs. 24 months	−0.064	1.316	0.199	−0.639	9.531	**0.001**
RNFL	0 vs. 6 months	−1.738	9.166	**0.001**	−5.030	20.137	**0.001**
	6 vs. 12 months	−0.741	5.680	**0.001**	−3.362	4.920	**0.001**
	12 vs. 18 months	−0.276	2.692	0.012	−4.476	37.065	**0.001**
	18 vs. 24 months	−0.144	2.468	0.020	−4.676	37.439	**0.001**
RNFL Sup	0 vs. 6 months	−1.594	9.114	**0.001**	−4.500	16.430	**0.001**
	6 vs. 12 months	−0.748	5.478	**0.001**	−4.055	28.439	**0.001**
	12 vs. 18 months	−0.277	2.445	0.021	−4.491	35.957	**0.001**
	18 vs. 24 months	−1.396	2.524	0.017	−4.858	52.242	**0.001**
RNFL Inf	0 vs. 6 months	−1.504	11.967	**0.001**	−4.511	25.876	**0.001**
	6 vs. 12 months	−0.749	7.200	**0.001**	−4.245	22.547	**0.001**
	12 vs. 18 months	−0.213	2.151	**0.040**	−4.542	35.663	**0.001**
	18 vs. 24 months	−0.225	2.633	**0.013**	−4.811	43.296	**0.001**
RNFL Nas	0 vs. 6 months	−1.595	8.190	**0.001**	−4.530	21.142	**0.001**
	6 vs. 12 months	−0.815	5.591	**0.001**	−4.160	27.324	**0.001**
	12 vs. 18 months	−0.176	2.215	**0.035**	−4.273	29.209	**0.001**
	18 vs. 24 months	−0.252	2.510	**0.018**	−4.865	38.397	**0.001**
RNFL Temp	0 vs. 6 months	−1.843	11.391	**0.001**	−4.538	21.436	**0.001**
	6 vs. 12 months	−0.726	5.899	**0.001**	−3.808	19.414	**0.001**
	12 vs. 18 months	−0.147	0.929	0.361	−4.319	24.487	**0.001**
	18 vs. 24 months	−0.170	2.255	**0.032**	−4.661	52.818	**0.001**
GCC	0 vs. 6 months	−1.532	10.125	**0.001**	−4.541	22.862	**0.001**
	6 vs. 12 months	−1.058	6.648	**0.001**	−4.552	20.706	**0.001**
	12 vs. 18 months	−0.162	2.290	**0.029**	−4.472	37.441	**0.001**
	18 vs. 24 months	−0.146	2.579	**0.015**	−4.928	39.883	**0.001**
GCC Sup	0 vs. 6 months	−1.149	7.218	**0.001**	−3.877	14.240	**0.001**
	6 vs. 12 months	−0.874	5.306	**0.001**	−3.987	18.143	**0.001**
	12 vs. 18 months	−0.270	2.573	**0.015**	−4.328	22.581	**0.001**
	18 vs. 24 months	−0.164	2.368	**0.025**	−4.785	40.536	**0.001**
GCC Inf	0 vs. 6 months	−1.329	2.467	**0.020**	−5.268	18.620	**0.001**
	6 vs. 12 months	−1.319	3.200	**0.003**	−5.101	11.989	**0.001**
	12 vs. 18 months	−0.383	2.604	**0.014**	−4.628	25.836	**0.001**
	18 vs. 24 months	−0.205	1.535	0.136	−5.026	31.681	**0.001**

Statistical analysis of the differences between evaluated parameters from baseline to 24 months’ follow-up. MD, mean deviation measured with standard automatic perimetry (SAP); RNFL, overall retinal nerve fiber layer thickness; RNFL Sup, superior retinal nerve fiber layer thickness; RNFL Inf, inferior retinal nerve fiber layer thickness; RNFL Nas, nasal retinal nerve fiber layer thickness; RNFL Temp, temporal retinal nerve fiber layer thickness; GCC: overall ganglion cell complex thickness; GCC Sup, superior ganglion cell complex thickness; GCC Inf, inferior ganglion cell complex thickness measured with ocular coherence tomography (OCT). p, Paired Student t-test significance (values < 0.05 in bold; values < 0.005 in bold on gray).

**Table 5:** Analysis of correlation between studied parameter values at baseline and variations of the same parameters during follow-up in both groups, in order to evaluate if baseline values influence the variations detected.

		Var 0−24%	
		Group A	Group B
MD	Pearson correlation	0.406	0.757
	Sig. (2-tailed)	**0.026**	**0.000**
	N	30	30
RNFL	Pearson correlation	0.374	0.540
	Sig. (2-tailed)	**0.042**	**0.002**
	N	30	30
RNFL Sup	Pearson correlation	−0.021	0.728
	Sig. (2-tailed)	0.912	**0.000**
	N	30	30
RNFL Inf	Pearson correlation	0.036	0.588
	Sig. (2-tailed)	0.849	**0.001**
	N	30	30
RNFL Nas	Pearson correlation	0.037	0.795
	Sig. (2-tailed)	0.846	**0.000**
	N	30	30
RNFL Temp	Pearson correlation	0.029	0.619
	Sig. (2-tailed)	0.880	**0.000**
	N	30	30
GCC	Pearson correlation	0.089	0.467
	Sig. (2-tailed)	0.639	**0.009**
	N	30	30
GCC Sup	Pearson correlation	0.067	0.421
	Sig. (2-tailed)	0.726	**0.021**
	N	30	30
GCC Inf	Pearson correlation	−0.239	0.336
	Sig. (2-tailed)	0.204	0.069
	N	30	30

Correlation analysis between the variations of the optic nerve parameters evaluated at the beginning of the study and the values observed after 24 months. MD, mean deviation measured with standard automatic perimetry (SAP); RNFL, overall retinal nerve fiber layer thickness; RNFL Sup, superior retinal nerve fiber layer thickness; RNFL Inf, inferior retinal nerve fiber layer thickness; RNFL Nas, nasal retinal nerve fiber layer thickness; RNFL Temp, temporal retinal nerve fiber layer thickness; GCC, overall ganglion cell complex thickness; GCC Sup, superior ganglion cell complex thickness; GCC Inf, inferior ganglion cell complex thickness measured with ocular coherence tomography (OCT). p, Pearson correlation significance (values < 0.05 in bold; values < 0.005 in bold on gray).

IOP value evaluations showed no intra-group significant difference during the follow-up in both groups.

## Discussion

Co-adjuvant neuroprotective treatment with oral CT in glaucoma patients has shown promising results, thanks to both neuroprotective and neuro-enhancement properties (Saver, 2008; Grieb, 2014; Wignall and Brown, 2014; Roberti et al., 2015). Previously published studies have evaluated the efficacy of CT on the visual field or electrophysiology (Virno et al., 2000; Parisi, 2005; Parisi et al., 2008; Ottobelli et al., 2013; Parisi et al., 2015). In this study, OCT has been utilized jointly with SAP to evaluate changes in RNFL and GCC, since, in recent years, this diagnostic approach has been proven to be more relevant in glaucoma diagnosis and management (Holló and Naghizadeh, 2015; Liu et al., 2015; Mwanza et al., 2015a; Mwanza et al., 2015b; Holló et al., 2016; Mwanza and Budenz, 2016). The differences observed in this study may depend on therapy adherence. In fact, if daily therapy is not well followed, it may produce spikes in IOP that lead to a worsening of retinal sensitivity (documented by SAP) and to a thinning of RNFL (measured with OCT). Patients enrolled in the study were always asked about therapy compliance, which proved to be appropriate. It is important to remember that patients whose tests showed IOP spikes at any of the follow-up checks, and who thus required additional therapy or surgery, were subsequently excluded from this study.

Data observed in this study suggest that CT has an effect in slowing the MD reduction measured with SAP after 18 months (**Figure 2**) of therapy and that this effect appears to be stable in the following 6 months (**Figure 2**, **Table 4**). It is interesting to note that this effect was not so evident at 6 and 12 months’ follow-up, thereby suggesting that this type of treatment needs time to show relevant clinical results.

Topical CT eye drops have been showed to have a positive effect in glaucoma patients, detected by pattern electroretinogram (PERG) and visual-evoked potentials (VEPs) (Parisi et al., 2015) after 4–6 months. This could suggest that electrophysiology devices are more sensitive in detecting CT-induced changes than are devices used in this study (SAP and OCT). In this study, oral medication has been investigated because it could be an easier option for patients, more for glaucoma patients who are usually older and have other different eye drops to take as medications, and instrumentations routinely used in glaucoma units confirmed the positive effect of this treatment. The advantage of using the oral solution is related to the compliance of the patients. To determine which between oral and topical CT has the best and faster effect is hard, because there are not so many studies investigating this topic, and none compared the two kinds of intake between them, but this could be the objective of a new prospective study. The important message of this study is that also oral CT has been proven to be effective in slowing optic nerve damage progression in glaucoma patients.

**Figure 5:**
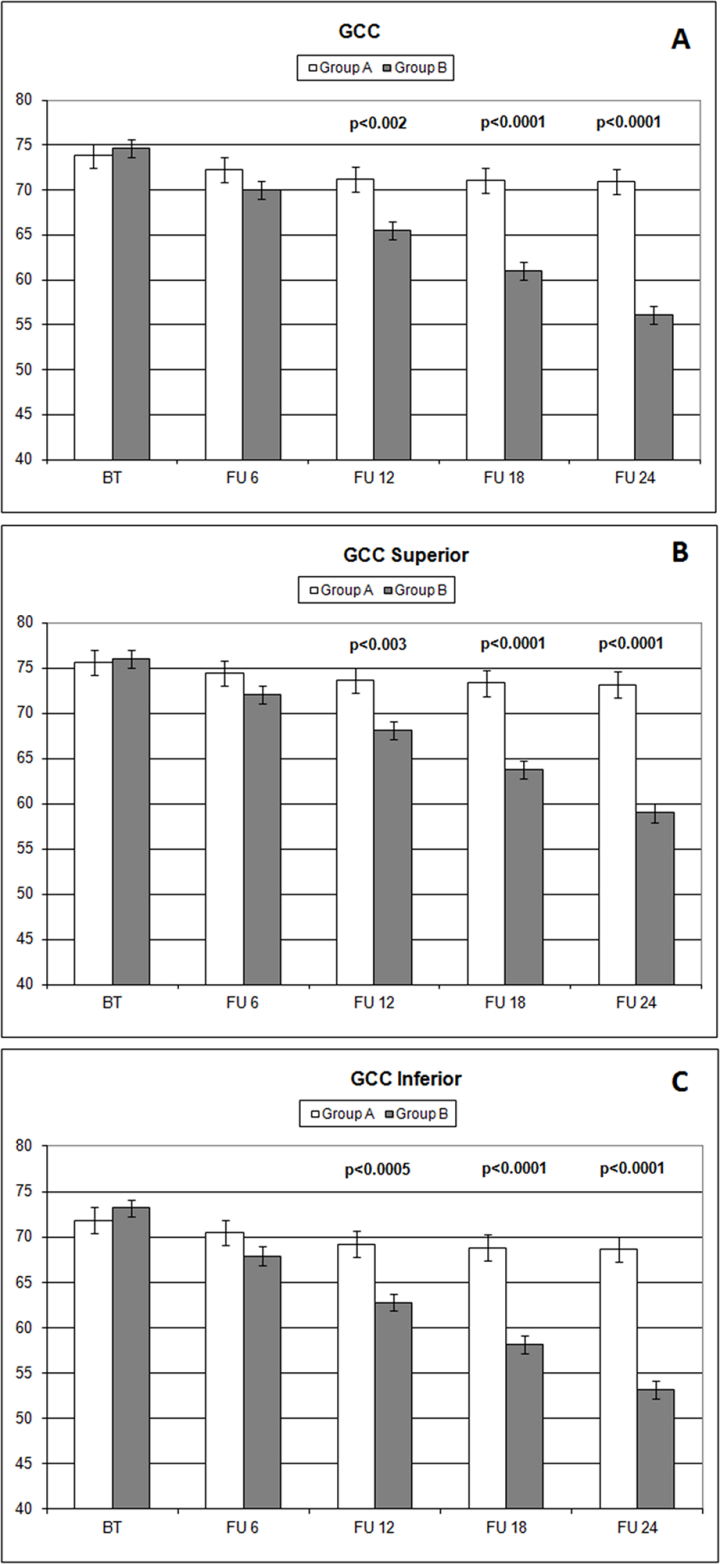
Comparison of overall ganglion cell complex thickness values and the ones measured (microns) in different sectors (**A**: overall; **B**: superior; **C**: inferior) with ocular coherence tomography (OCT), on the vertical axis, in patients assuming citicoline (white column) and in patients not undergoing therapy with citicoline (grey column), before starting therapy (BT), at 6 months’ follow-up (FU 6), at 12 months’ follow-up (FU 12), at 18 months’ follow-up (FU 18), and at 24 months’ follow-up (FU 24). *p* values mean significant differences (*t*-test).

The analysis of overall RNFL changes indicated that the overall thinning is significantly slower after 12 months’ follow-up (**Figures 3** and **4**) and it appears to be stable in the following 12 months (**Figures 3** and **4**; **Table 4**). Overall, superior and inferior GCC thickness showed similar behaviors during the time of observation (**Figure 5**, **Table 4**). Interestingly, temporal and nasal RNFL thickness of group A showed significantly (*p* < 0.05) higher values, than did those of group B, after 6 months of treatment. According to these data, OCT seems to be more sensitive in detecting the neuroprotective effect provided by oral CT treatment, highlighting it 6 months before SAP. In particular, temporal and nasal RNFLs show higher sensitivity than all other parameters provided by OCT. Moreover, in this study, no significant correlation was observed between SAP and OCT parameters evaluated at baseline and their variations during follow-up (**Table 5**). This is a very interesting outcome, because, if confirmed by further studies, it suggests that CT therapy is useful at every stage of glaucoma.

One of the limitations of this study consists in the heterogeneity of the glaucoma stage in the patients enrolled, since the study was not limited to middle-stage glaucoma. On the other hand, this characteristic provides a more realistic representation of the population of patients usually referring to glaucoma units. The data analyzed in this study come from the evaluations routinely performed in the glaucoma unit. It is important to note that this is a prospective, randomized, comparative study and that this may, therefore, be considered a strength of this analysis. In order to improve this study, it would be useful to add new optic nerve testing devices to this protocol, and such devices will certainly be added to any future studies by this group.

Another limitation of the study is the small sample analyzed; it is very difficult to collect data on patients with the required characteristics in one glaucoma unit and evaluate them for 2 years. It is hoped that this study will encourage future research with larger study populations.

The findings of this study agree with the ones published by Parisi et al. and Ottobelli et al. (Parisi et al., 2008; Ottobelli et al., 2013; Parisi et al., 2015), but our work provides a deeper analysis of optic nerve structures, by exploring the variation of RNFL and GCC. Previous studies on CT therapy effects have often been hard to compare due to the differences in the dosage, means of administration, and duration of therapy (Virno et al., 2000; Grieb and Rejdak, 2002; Rejdak et al., 2003; Parisi, 2005; Foganolo and Rossetti, 2011; Grieb, 2014; Roberti et al., 2015; Tian et al., 2015). This study, prescribing a new formulation specifically designed for glaucoma patients, is able to demonstrate data safely and easily reproducible in every kind of practice. Frequently, one of the most important factors causing low compliance in this kind of treatment is the CT administration mode (Grieb and Rejdak, 2002; Grieb, 2014). In contrast, here, the vial, following the manufacturer regimen, was well tolerated by the patients.

This study highlights a very interesting aspect of oral CT treatment: The effects are not visible rapidly. More than 1 year is needed to detect significant changes in MD (**Figure 2**); therefore, physicians should support the idea of extending this therapy for longer than they usually do. OCT is able to highlight significant variations faster than other devices (**Table 4**), but it still requires months-long period. In conclusion, although these results need to be confirmed by further studies with a longer follow-up period and performed on a larger population, the data recorded confirm that CT administrated as an oral solution is effective in slowing the progression of POAG at different stages of the disease.

## Data Availability Statement

The datasets for this manuscript are not publicly available because privacy of the involved subjects. Requests to access the datasets should be directed to Michele Lanza, mic.lanza@gmail.com.

## Ethics Statement

The study was performed in accordance with the ethical standards stated in the 1964 Declaration of Helsinki and approved by the local clinical research ethics committee (University of Molise); informed consent was obtained from all subjects after a detailed description of the aim work and the procedure used. Investigations have been conducted in accordance with Good Clinical Practice (GCP).

## Author Contributions

ML conceived and designed the study and undertook data collection; UC undertook data collection and statistical analysis; LM performed visits and wrote the manuscript; MS wrote the manuscript and undertook supervision; SB performed visits and undertook data collection; CC conceived and designed the study, performed visits, undertook supervision, and gave final approval.

## Conflict of Interest

The authors declare that the research was conducted in the absence of any commercial or financial relationships that could be construed as a potential conflict of interest.
